# The *Steppengrille* (*Gryllus spec./assimilis*): Selective Filters and Signal Mismatch on Two Time Scales

**DOI:** 10.1371/journal.pone.0043975

**Published:** 2012-09-07

**Authors:** Matti Michael Rothbart, Ralf Matthias Hennig

**Affiliations:** Behavioural Physiology, Department of Biology, Humboldt-Universität zu Berlin, Berlin, Germany; Utrecht University, The Netherlands

## Abstract

In Europe, several species of crickets are available commercially as pet food. Here we investigated the calling song and phonotactic selectivity for sound patterns on the short and long time scales for one such a cricket, *Gryllus* spec., available as “*Gryllus assimilis*”, the *Steppengrille*, originally from Ecuador. The calling song consisted of short chirps (2–3 pulses, carrier frequency: 5.0 kHz) emitted with a pulse period of 30.2 ms and chirp rate of 0.43 per second. Females exhibited high selectivity on both time scales. The preference for pulse period peaked at 33 ms which was higher then the pulse period produced by males. Two consecutive pulses per chirp at the correct pulse period were already sufficient for positive phonotaxis. The preference for the chirp pattern was limited by selectivity for small chirp duty cycles and for chirp periods between 200 ms and 500 ms. The long chirp period of the songs of males was unattractive to females. On both time scales a mismatch between the song signal of the males and the preference of females was observed. The variability of song parameters as quantified by the coefficient of variation was below 50% for all temporal measures. Hence, there was not a strong indication for directional selection on song parameters by females which could account for the observed mismatch. The divergence of the chirp period and female preference may originate from a founder effect, when the *Steppengrille* was cultured. Alternatively the mismatch was a result of selection pressures exerted by commercial breeders on low singing activity, to satisfy customers with softly singing crickets. In the latter case the prominent divergence between male song and female preference was the result of domestication and may serve as an example of rapid evolution of song traits in acoustic communication systems.

## Introduction

The calling songs of many species of crickets and bushcrickets transport information on two time scales ([1], [2]). For crickets, the pulse period, often described as pulses per second, is usually limited to a time scale below 100 ms. Pulses are grouped into trills or chirps that are emitted at a longer time scale of more than 100 ms. ([3], [4]). These cues are commonly used by taxonomists which use three song features for a successful discrimination of species: pulses per second, number of pulses per chirp and chirp rate ([4]). It is less clear whether these cues are also relevant for female crickets to discriminate conspecific from heterospecific songs. Although the band-pass properties of the pulse filter were described for several species ([5], [6], [7], [8]), the selectivity of females on the long time scale is known in detail only for few species ([9], [10], [11], see [12] for bushcrickets). Here we chose to examine the preferences of females of a commercially available cricket in Europe, the *Steppengrille (Gryllus spec./assimilis)*. This species is in the same genus as the well investigated *G. bimaculatus* and shares the basic properties of the calling song, that is pulses grouped into short chirps. By a description of the transfer function and of the profiles of the pulse and the chirp filter we aimed to compare the characteristic features of the receiver, the females, between the two species. We also determined the values and coefficients of variation for calling song parameters, to test whether a conceivable mismatch with receiver preferences may originate from directional selection on male traits by females.

## Materials and Methods

### Animals

Males and females of the *Steppengrille (Gryllus spec./assimilis)* were obtained as nymphs from a commercial supplier. Males and females were raised to adulthood separately. Virgin females were tested from the seventh day after their final moult.

### The origin of the *Steppengrille (G. spec./assimilis)* in Europe

Animals were caught in late 1970 in Ecuador and identified as *Gryllus* spec. possibly *Gryllus assimilis*. All present European stocks originated from Grigfarm, CH, and were distributed by commercial suppliers as *Gryllus assimilis*, the steppe dwelling cricket (*Steppengrille*; Rotter, personal communication). The *Steppengrille* was easy to raise in culture and soon became a popular species among customers for feeding their pets. The crickets were convenient to keep at home for customers, mostly because of their soft song (Rotter, Hildner and Tindner personal communication). The *Steppengrille* was confirmed as a *Gryllus* by David Weissman (personal communication) after inspection of several specimens, but does not belong to the *G. assimilis* group described previously from Central America ([13]). Morphological measures of the *Steppengrille* are given in table 1.

**Table 1:** Morphological measures of the *Steppengrille (G. spec./assimilis)*.

hind femur	Pronotum	Cephalon	wing	file
length [mm]	width [mm]	length [mm]	width [mm]	width [mm]	length [mm]	width [mm]	width w/o side	length [mm]	teeth	teeth/mm
10,05	3,32	3,45	5,49	4,95	13,18	6,93	4,63	3,44	137,1	45,80
0,64	0,25	0,28	0,43	0,39	1,15	0,46	0,33	0,32	16,7	3,67

n = 20, data as mean and standard deviation.

### Song recording and analysis

Individual male crickets were kept in jars (50×50×80 mm) with a microphone (TCM 141, Conrad) placed above. The containers of up to 16 males were simultaneously placed in a shelf arranged in 4 vertical rows with 4 boxes each. Sound transmission from singing males to their neighbours was minimized by insulating foam. Songs of singing males were sampled to hard disk by an AD-board (PCI-6221, National Instruments, Texas) at 100 kHz. A standard personal computer was programmed to start sampling the songs of single males upon detection of singing activity. During each recording session a maximum of 5 continuous song sequences lasting 20 s were recorded for each individual male. Temperature for song recordings was between 26 and 29°C. Temporal measures of song recordings were corrected to 25°C ([14])

Song analysis was performed offline and the peak of the carrier spectrum as well as temporal measures of the song envelope computed. For the latter, the song signal was rectified by calculation of the RMS with time window of 4 ms ([15]). Pulse and pause durations were obtained by applying a threshold function at 10–15% after normalization of the song envelope to its maximal value. From the consecutive series of pulse and pause durations, pulse periods and pulse duty cycles (pulse duration divided by pulse period) were computed. Groups of pulses followed by a long pause (larger than 60 ms) were assigned as chirps. For chirps the number of pulses, the chirp duration and chirp pause as well as the chirp duty cycle (chirp duration divided by chirp period) was calculated.

### Phonotaxis experiments

Behavioural tests were performed on a locomotion compensator (Kramer treadmill; for details see [16]) in an anechoic chamber at 25±2°C. Experiments were conducted in the dark except for the infrared light used to monitor the movements of the cricket. Crickets were placed on top of a sphere, free to walk but kept in place by compensatory sphere rotations, while song models were presented from loudspeakers in their horizontal plane. The rotations of the sphere were monitored by displacement transducers. After sampling their output signal by an AD-board (PCI-6221, National Instruments, Texas, 10 kHz), the walking distance and angle of orientation towards the loudspeaker were calculated (see below).

### Stimulus generation and presentation

Song models were generated digitally by multiplication of a given signal envelope with a sine wave (5.0 kHz) using LabView Software (National Instruments, Texas). Envelopes of sinusoidal stimuli were constructed by sine waves at frequencies between 1 Hz and 50 Hz.

**Figure 1:**
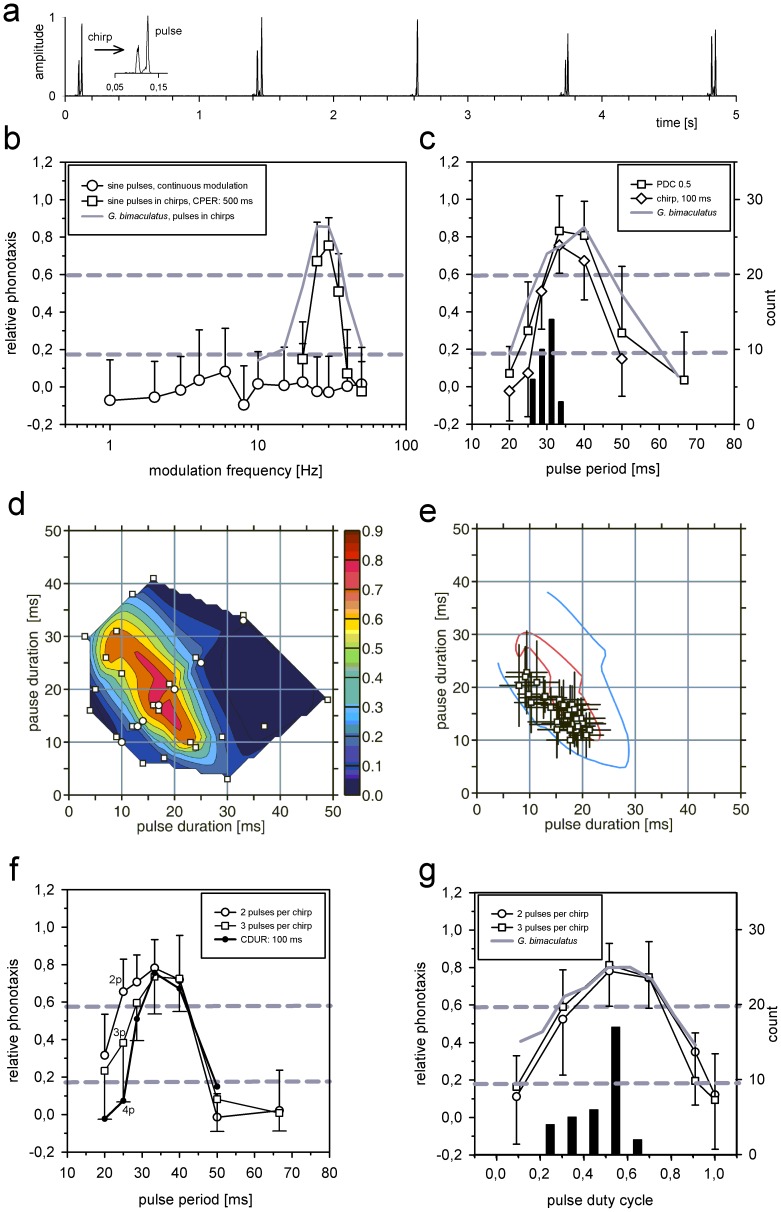
Properties of the pulse filter of females of *Steppengrille G. spec./assimilis* in comparison with song data. **a** Envelope of the song pattern of a male (inset: expanded chirp). **b** Transfer function of the phonotactic response for females to envelope patterns with different modulation frequencies. Presented stimuli consisted of continuous, sinusoidal modulations (open circles) or groups i.e. chirps of sinusoidal pulses (open squares, presented with a chirp duration of 100 ms and chirp period of 500 ms). Envelopes of all stimuli were filled with a carrier of 5.0 kHz. For comparison the pulse filter of the cricket, *G. bimaculatus*, is shown (gray line). **c** Selectivity of females for stimuli with rectangular (open squares) or sinusoidal pulses (open diamonds) presented in chirps as in **a** (pulse duty cycle of 0.5, chirp duration 100 ms, chirp period: 500 ms; for comparison the pulse filter of the cricket, *G. bimaculatus*, is also shown (gray line)). Histogram shows the distribution of pulse periods measured from individual male songs (c.f. table 2). **d** Response profile of females for patterns with different combinations of pulse and pause durations (stimuli consisted of chirps of 100 ms duration presented at a chirp period of 500 ms). Open squares indicate test stimuli with rectangular pulses, open circles refer to stimuli with sinusoidal pulses. **e** Pulse and pause durations (mean and standard deviation) from song patterns of individual males plotted within the response profile shown in **d**. Response ranges from **d** are shown for values of relative phonotaxis at 0.25 (blue line) and 0.75 (red line). **f** Responses of females to pulse patterns with different duty cycles tested with stimuli that contained only 2 (open circles) or 3 pulses per chirp (open squares) presented at a constant pulse period of 33 ms. For comparison responses of *G. bimaculatus* are indicated (gray line). The distribution of duty cycles measured from individual song data are shown as black bars. **g** Responses of females to pulse patterns with only 2 (open circles) or 3 pulses per chirp (open squares) presented at different pulse periods. Chirp period was kept constant at 500 ms. Responses to patterns with a constant chirp duration are shown for comparison (black circles, from **c**). For a pulse period of 25 ms the number of pulses in a chirp (2p, 3p and 4p) are indicated. Data is given as mean values and standard deviation. Gray stippled lines in panels **b, c, f, g** mark levels of significance, see methods.

The envelope for song models with rectangular pulses and pauses was generated with rise and fall times of 1 ms. Pulsed stimuli were either continuous or contained groups of pulses, mimicking a chirp of the *Steppengrille G. spec. (assimilis)* (c.f. Fig. 1a). Acoustic stimuli were stored on disk and broadcast after DA-conversion (16-bit resolution, 100 kHz sampling rate; PCI-6221 National Instruments, Texas) via one of two loudspeakers (Piezo Horntweeter PH8) separated by an angle of 90°. Signal amplitude was calibrated with a condenser microphone (Bruel & Kjaer Type 4133) and a measuring amplifier (Bruel & Kjaer Type 2231). Sound measurements were obtained 0.5 cm above the sphere, with the microphone directed towards the loudspeaker. Sound intensities were 80±2 dB SPL (re. 2×10^−5^ Pa, fast reading).

Temporal measures of patterns were obtained with a threshold at 15% of the maximum that returned the values for pulse and pause duration. From these values period duration and duty cycle (pulse duration divided by period duration [5], [17]) were calculated.

### Stimulus protocol and data evaluation

Acoustic stimuli were presented for 100 s from each loudspeaker consecutively. A control stimulus, similar to the calling song of males (3 sinusoidal pulses at a pulse period of 33 ms and a chirp period of 500 ms), was presented at the beginning and also the end of each session in order to control for a change of motivation (positive control). Female crickets were tested with 4 to 7 test stimuli in each session (25 to 40 minutes). Further controls included the presentation both of continuous unmodulated tones at 5.0 kHz as well as silent intervals for 100 s, in order to obtain measures for baseline activity of individual females (negative control). Before the presentation of a stimulus from a respective loudspeaker a silent break of 10 s was maintained, in order to minimize possible hysteresis effects from the previous pattern (see [16] and [18] who reported a time constant of 5 to 7 s for the decay of phonotactic orientation after presentation of an attractive signal).

For each walking track the walking distance, vector length and angle towards the loudspeaker was calculated and referenced to the same measures of the walking track obtained for the initial control stimulus (see [19] for details). For crickets, this measure of relative phonotaxis indicates positive or negative phonotaxis at values greater than 0.8 or lower than −0.8, respectively, and served as valid indicator for the attractiveness of a stimulus ([19], [7]). Values around zero indicated no preferred orientation and were usually the result of circular walking tracks.

Test sessions in which the response to the final positive control was more than 20% below that of the first control or in which the responses to continuous tones or silent intervals serving as negative controls was higher than 0.2 were excluded from the data set. Data is given as mean values and standard deviation.

**Figure 2:**
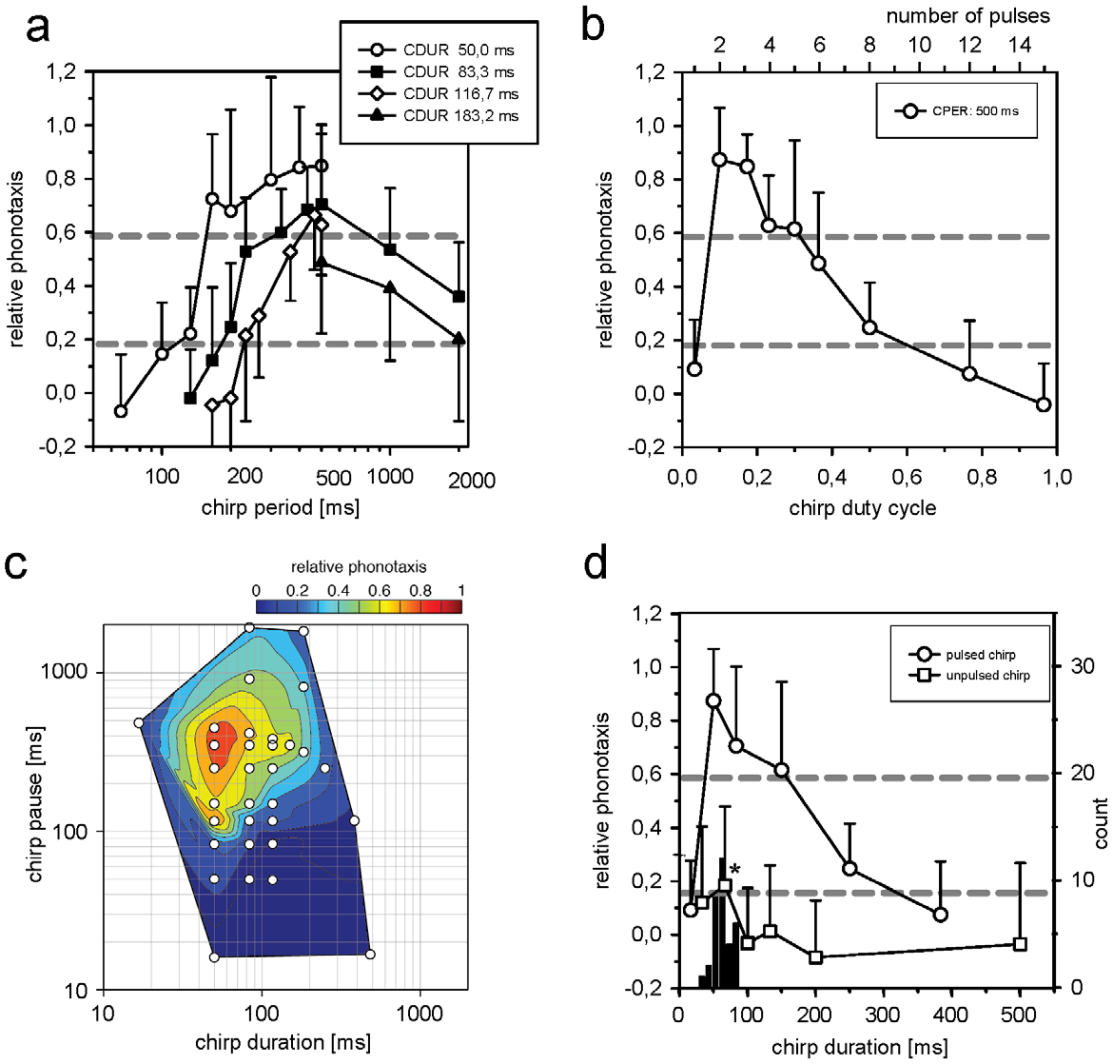
Properties of the chirp filter of females of the *Steppengrille G. spec./assimilis* in comparison with song data. **a** Phonotactic response of females to patterns with different chirp periods for sets of stimuli with different chirp durations (CDUR). **b** Phonotactic response of females to patterns with different duty cycles of the chirp pattern (chirp period was kept constant at 500 ms; the top axis refers to the number of pulses in a stimulus). **c** Response profile of females for patterns with different combinations of chirp duration and chirp pause on a double logarithmic scale (tested stimuli are indicated by open squares, all stimuli contained pulse periods of 33 ms). **d** Phonotactic response of females to patterns with different chirp durations, but constant chirp periods of 500 ms (open circles; pulse period was kept constant at 33 ms). Scores of relative phonotaxis in response to different chirp durations without modulation by pulses are shown by open squares. Histogram shows the distribution of chirp durations measured from individual male songs (c.f. table 2). The response marked by an asterisk was significantly different from the silent (p<0,05), but not from the continuous tone control. Data is given as mean values and standard deviation. Gray stippled lines in panels **a, b, d** mark levels of significance, see methods.

Statistical analysis was performed with raw data, that is the value of relative phonotaxis obtained for one female for a particular test or control stimulus. For a test series from 12 to 16 different females were tested. In most cases but not all the scores of relative phonotaxis were normally distributed. Therefore a non-parametric test was chosen (Friedman Test (Nonparametric Repeated Measures ANOVA) with Dunn post-test in GraphPad Software, California) considering multiple tests of individual females in different test series. For all test series the relative phonotactic scores for the positive control (first and last control pattern in a test series) were not significantly different from one another. Equally, there was no significant difference between two negative controls (the silent and continuous tone control). In Figs. 1 and 2 the scores obtained from test patterns were compared to both the positive and the negative control stimuli. Three classes of attractiveness emerged for all test patterns. The scores for test patterns were either attractive and not significantly different from the positive or unattractive and not significantly different from the negative control. Test patterns that differed significantly from both control types indicated intermediate attractiveness. The margins for the three classes were marked by stippled lines in Figs. 1 and 2. The calculation of preference profiles from data in Figs. 1d and 2c was based on a triangulation and cubic interpolation ([20]).

The performed experiments complied with the “Principles of animal care”, publication No. 86-23, revised 1985 of the National Institute of Health, and also with the current laws of Germany.

## Results

### Analysis of male songs

The songs of the *Steppengrille G. spec./assimilis* were analyzed for the spectral content of the carrier and several variables that characterized the envelope of the song pattern (table 2). The carrier frequency revealed a mean value of 4.98 kHz and the coefficient of variation between males was at 5.8%, indicating only little variability between males. On average males produced 2 to 3 pulses per chirp, but it was not uncommon to observe series of chirps with only single sound pulses in a recording sequence (Fig. 1a). The pulse period was at 30.2 ms and revealed the lowest coefficient of variation of all envelope variables (table 2). The duty cycle of the pulse pattern was close to 0.5 and thus the pulse durations were on average as long as the pause durations. Chirps were separated by rather long pauses of more than 2 s (table 2).

**Table 2:** Spectral and temporal measures of the songs of the *Steppengrille (G. spec./assimilis)*, data from 34 males.

	carrier	pulse pattern		chirp pattern	
	frequency [kHz]	pdur [ms]	ppau [ms]	pper [ms]	pDC	nr,P	Cdur [ms]	Cpau [ms]	Cper [ms]	CDC
mean	4,98	16,00	15,77	30,22	0,48	2,52	64,40	2274,0	2338,4	0,03
sd	0,29	3,62	4,81	3,98	0,11	0,38	12,52	608,1	612,6	0,01
CV, inter	5.8%	22.6%	30.5%	13.2%	22.9%	15.0%	19.4%	26.7%	26.2%	33.0%
CV, intra	n.a.	26.0%	27.0%	14.0%	27.0%	37.0%	44.0%	27.0%	26.0%	45.0%

Values were corrected to a temperature of 25°C.

Explanation of terms: pdur: pulse duration, ppau: pause duration, pper: pulse period, pDC: pulse duty cycle (pdur/pper), nr,P: number of pulses per chirp, Cdur: chirp duration, Cpau: chirp pause, Cper: chirp period, CDC: chirp duty cycle (Cdur/Cper). CV, inter: coefficient of variation between individuals (sd/mean), CV, intra: mean of coefficient of variation measured for individual males. On average 138 pulses (range: 15–483) and 58 chirps (range: 7–186) were analyzed per individual male song.

### Properties of the pulse filter of females

In a first test series the transfer function for continuous sinusoidally modulated pulses was determined, in order to examine whether continuous stimuli may already elicit positive responses from females of the *Steppengrille G. spec./assimilis* at particular modulation frequencies (Fig. 1b). None of these test stimuli elicited a response significantly different from the negative controls i.e. silence and continuous tone (Fig. 1b, open circles). However, to pulse frequencies of 30 Hz females responded with high phonotactic scores, if sinusoidal pulses were grouped into chirps with a duration of 100 ms and presented at a chirp period of 500 ms (Fig. 1b, open squares). A comparison with the equivalent pulse filter response of females of the cricket *G. bimaculatus* (Fig. 1b, gray line, [7] Hennig 2009) showed the same best modulation frequency for both species, however, the pulse filter of *G. assimilis* was more sharply tuned. In order to compare the preference of females for pulse frequencies of 30 Hz with the corresponding pulse period produced by males, the results of two test series with rectangular pulse patterns are shown together with the pulse period distribution on a linear time scale in Fig. 1c. On average males produced shorter pulse periods than were preferred by females and only few males revealed pulse periods longer than 30 ms. The signal distribution of the sender and the preference function of the receiver indicated a mismatch.

In order to better characterize the preference function of females with respect to pulse and pause durations of test patterns, a range of different tests stimuli with different combinations was tested. Since stimuli with a chirp period of 500 ms and a chirp duration of 100 ms were accepted best by females (open squares in Fig. 1b), variations of the pulse pattern were presented with these temporal values of the chirp pattern (see also next section). The response array in Fig. 1d revealed that the preference of females extended along a range of pulse periods at 30 ms to 35 ms. Within this range different combinations of pulse and pause durations were accepted over a wide range. In Fig. 1e the pulse and pause durations of individual males confirmed the mismatch observed in Fig. 1c, since the data of most males gathered at shorter pulse periods (in Fig. 1e the range of female preferences from Fig. 1d were indicated by phonotactic scores of 0.25, blue, and 0.75, red). The variation of pulse durations from 8–22 ms and pause durations from 10–23 ms in male songs was not due to changes in threshold used for the analysis (see methods), since variations in threshold between 10 and 15% accounted only for differences of 1–2 ms. A mismatch for the duty cycle of the pulse pattern was not observed (Fig. 1f), since males revealed pulse duty cycles between 0.3 and 0.7 that were well accepted by females (data from *G. bimaculatus* was included in Fig. 1f for comparison). Note that for the measurement of the response curves of females in Fig. 1f, a fixed pulse period of 33 ms was used and the number of pulses within a chirp was kept constant at either 2 or 3 pulses. Since for these latter response curves there was no apparent difference between chirps with only few pulses, the response function for pulse period was also determined with a fixed number of pulses (Fig. 1g). The best pulse period and the drop of responses to longer pulse periods was identical for test patterns with 2 or 3 pulses (Fig. 1g). However, at short pulse periods below 30 ms the response curves of females diverged. Stimuli with only 2 pulses were accepted better than stimuli with more pulses (see symbols marked with the respective pulse number: 2p, 3p, 4p in Fig. 1g at a pulse period of 25 ms). In Fig. 1g data from tests with a constant chirp duration of 100 ms and thus more pulses at shorter pulse periods were also included. This reduction in the attractiveness of patterns with more pulses, and thus pulse periods, was most pronounced for shorter pulse period at which males produced their songs (Fig. 1c, table 2).

### Properties of the chirp filter of females

Besides the information transported on the short time scale by the pulse period, crickets differ also in the rate at which chirps are produced. The tests in Fig. 1b had already shown that a continuous series of pulses at the preferred pulse period of 33 ms without the chirp structure, which was equivalent to a modulation frequency of 30 Hz, was not sufficient to elicit positive responses. During initial, preliminary tests in which the presented patterns were similar to that of the calling songs of males with chirp durations of 100 ms and chirp periods of 2000 ms female cricket failed to respond well. In order to examine the selectivity of female crickets on the long time scale, we systematically varied the temporal variables of the chirp pattern (chirp duration, chirp pause and chirp period) in further tests and kept the pulse period at the known optimum of the response at 33 ms (c.f. Fig. 1c).

In a first series of tests, the chirp duration was kept at a fixed value and the chirp period was varied by changing the duration of the chirp pause (Fig. 2a). If chirp period was the crucial variable for recognition of the correct chirp pattern, the response scores for different chirp durations but the same chirp period should fall on top of each other. That, however, was not observed (Fig. 2a): at chirp periods below 500 ms the responses for different chirp periods were dependent on the chirp duration used. For shorter chirp durations also shorter chirp periods were accepted (open circles, black squares in Fig. 2a). Best responses for the chirp period extended over a range from 200 ms to 500 ms. At a chirp period of 500 ms the shorter chirp durations exhibited higher scores than longer chirp durations. At longer chirp periods (1000 and 2000 ms) the scores of relative phonotaxis declined for chirp durations of 83.3 ms (3 pulses) and 183.2 ms (6 pulses, Fig. 2a). At chirp periods of 2000 ms the response of females was weak, although the male signal on average contained longer chirp periods (table 2). The observed shift of the preferred chirp period in dependence of the chirp duration suggested the chirp duty cycle as a cue for positive responses.

In a further test series, the duty cycle was varied systematically by variation of the chirp duration at a constant chirp period of 500 ms (Fig. 2b). The best responses were obtained for duty cycles between 0.1 and 0.2, while scores of relative phonotaxis strongly decreased at higher duty cycles. At the shortest duty cycle the response of females dropped strongly, since this stimulus contained only single pulses and thus no information about pulse periods below 100 ms (Fig. 2b, upper x-axis). These results account for the failure to observe an increased response at low modulation frequencies in the transfer function (Fig. 1b), since in these tests the duty cycle of the sinusoidal modulation was at 0.5 and thus beyond the accepted range (c.f. Fig. 2b). By the combination of different chirp durations and pauses it was possible to construct a response array for temporal cues of the chirp filter as before for the properties of the pulse filter (Fig. 1c). Fig. 2c showed two major cues for attractiveness of a song pattern on the long time scale: besides a limited chirp duty cycle (0.1–0.3, Fig. 2b) a range of chirp periods (200 to 500 ms, Fig. 2a) was accepted by females. Since females in previous tests had responded to chirps build from only two pulses (Fig. 1g), the question arose, whether the pulse modulation within a chirp was an important cue for positive phonotaxis or whether activation of the chirp filter was possible by simply choosing the correct chirp variables. Females had responded at a chirp period of 500 ms to most chirp durations with medium to high scores of relative phonotaxis (Fig. 2a). Females were tested with patterns at a chirp period of 500 ms in which the chirp duration was varied. In one series the chirp pattern contained a modulation by pulses at the attractive pulse period of 33 ms and in a second series the chirps were not modulated by pulses (Fig. 2d). Test patterns with a pulse modulation were most attractive, if the chirp duration was between 50 ms and 100 ms (open circles in Fig. 2d) at which males also produce their chirps (table 2). Single pulses did not elicit positive responses as observed before (black cross in Fig. 2a). Unmodulated chirp patterns were generally not attractive, however, at a duration 67 ms (2 pulses) a small increase in the scores of relative phonotaxis was observed, that was significantly different from the negative silent control (asterisk in Fig. 2d).

## Discussion

Generally, sender and receiver characteristics in communication systems are expected to match or to show at least an overlap of their respective distributions ([21]). Here we compared the properties of the male songs of the *Steppengrille (G. spec./assimilis)* with the selective preferences of the receiver that operates on two different time scales, the pulse filter and the chirp filter. For both filters very selective properties were observed and on both time scales a mismatch of particular cues was found (Fig. 1c, e; Fig. 2b, table 2).

### Receiver properties: selective tuning on two different time scales

The song of the *Steppengrille (G. spec./assimilis)* conformed to the pattern of many species of crickets worldwide and is not unlike that of the well investigated model system of the cricket *G. bimaculatus*: sound pulses are grouped into chirps that are transmitted at a lower rate. The receiver properties of females of the *Steppengrille (G. spec./assimilis)* corresponded to the basic arrangement of two filters for different time scales that is known from *G. bimaculatus* (Figs. 1d, 2c, [7], [9], [11]). However, there were several remarkable differences between these two species. As in *G. bimaculatus* the pulse filter emerged as a filter for pulse period limited by low and high duty cycles (Fig. 1c, d). While the preference function for pulse duty cycles and the best modulation frequency of the pulse filter were virtually identical with those of *G. bimaculatus*, the pulse filter of the *Steppengrille* was more selective and sharply tuned (Fig. 1b, c, [7]). Since females accepted patterns composed from sinusoidal and rectangular pulses equally well, the temporal detail of the pulse envelope is apparently not relevant for the computation of the pulse period ([15]). Nevertheless, even for patterns composed of only two pulses which provided only a single pulse period per chirp for sensory processing, the filter tuning remained narrow and became wider only at shorter but not longer pulse periods (Fig. 1g).

The transfer function for continuous modulation frequencies also differed between the two species: females of *G. bimaculatus* revealed increased responses at the best modulation frequencies of the pulse and chirp filter which was not observed for the *Steppengrille*, although the combined activation of both filters elicited a strong response (Fig. 1b, [7]). Differences between the two species were also obvious on the long time scale on which the chirp filter operates. Females of *G. bimaculatus* accepted a wide range of chirp patterns over a broad range of duty cycles ([9], [11]). By contrast, females of the *Steppengrille* accepted only short chirp durations below 100 ms (Fig. 2b, d) and revealed a high selectivity for chirp duty cycles below 0.25 (Fig. 2b, c). At present the physiological mechanisms in the auditory pathway that are responsible for the observed selectivity on both time scales remain to be explored. While the low responses at a modulation frequency of 2 Hz, equivalent to a chirp period of 500 ms, to continuous pulse series can be assigned to the inappropriate duty cycle of these patterns presented for the measurement of the transfer function (Fig. 1b, 2b), the interaction of both filters in the *Steppengrille* seemed to be more selective and stringent than in *G. bimaculatus* ([9], [11]). While in the latter a weak activation of the output is observed upon activation of only one filter, this was not observed for the *Steppengrille*, although there was a weak response, if an unmodulated chirp with a duration of less than 100 ms was presented (asterisk in Fig. 2d). From an evolutionary point of view, high selectivity suggests strong selection pressures on the receiver to correctly discriminate the songs of its own species from others ([22]). Notably, *G. bimaculatus* is one of overall few species crickets in Europe and likely faces little selective forces from other crickets with similar song patterns which may explain the broad tuning of both filters ([23]).

### The origin of the Steppengrille and its competitive surround

In contrast to the situation in Europe, crickets in North and Central America are confronted with strong competition between species ([24]). Recent data suggested that crickets in Central America not only compete for narrow transmission channels in the carrier frequency band between 3–9 kHz ([25]), but also for coding space in the temporal domain ([13]). The gross morphological appearance of the *Steppengrille* corresponds to that of the genus *Gryllus*, but not to the *G. assimilis*-group known from Central America (David Weissman personal communication). Furthermore, the recorded song pattern with short chirps composed from few pulses (less than 3 versus 8–20 for the *G. assimilis*-group) and relatively low pulse rates (30 p/s vs. 40–100 p/s) provided evidence against a close relationship between these species with the *Steppengrille* (table 2, [13]). Common to these species is apparently only the long chirp period of 2 s. Although the taxonomic identification of the *Steppengrille* remains elusive at present, its likely origin from Ecuador indicates high competition between species of crickets for signal space and likely explains the selective filter properties of females observed here on both time scales (Figs. 1, 2).

### Sender and receiver: match and mismatch

Directional selection due to sexual selection by female choice is a well known cause for a mismatch between sender and receiver ([21], [26], [27]). A signal property under directional selection is likely to exhibit higher variances than under stabilizing selection. For these differences, Gerhardt ([22]) coined the terms static and dynamic properties. For the *Steppengrille* several mismatches were observed on the short and long time scale (Fig. 1c, e, Fig. 2b).

While the duty cycle of the pulse pattern of the *Steppengrille* matched the response function of females, the pulse periods clearly lied at the edge of the female preference (Fig. 1c, g). Nevertheless, the variability between males as measured by the coefficient of variance was the lowest observed for any temporal cue (table 2). At present it remains unclear, whether there is indeed directional selection on pulse period despite its low variance and which physiological limitation may prevent males from singing with longer pulse periods as preferred by the females (Fig. 1c). It appears unlikely that the production of lower pulse rates is limited by energetic constraints ([28]). Also a neuronal constraint for a central pattern generator driving the wing movements is hard to conceive ([29]). However, Weissman et al. [13] reported a relationship between the carrier frequency and the pulse rate and thus pulse period, since at lower carrier frequencies males across populations of the *G. assimilis*-group produced higher pulse rates and thus shorter pulse periods. With a pulse rate of 30 pps and a carrier frequency of 5.0 kHz (table 2), the *Steppengrille* matched the described trend at higher carrier frequencies (c.f. Fig. 5 in [13]). This linkage of neuronal and morphological characters could result from pleiotropic effects or gene coupling such that at a given carrier frequency only a limited shift of the pulse rate is possible. Interestingly, males may reduce the selection by the sharply tuned filter on the pulse period by a reduction in the number of pulses per chirp (Fig. 1c, f). Although females prefer short chirps, these also have the effect that the width of the pulse filter at short pulse periods is broadened (Fig. 1g).

On the long time scale, there was an obvious mismatch between a chirp period of 2 s produced by males (table 2) and the preference by females tuned to a chirp period of 200–500 ms (Fig. 2c). The coefficient of variation for chirp period (26.2%) was higher than for pulse period (13.2%, table 2). Since Gerhardt [22] observed CVs of more than 100% for cues under directional selection, the low values observed here are not strong indicators for directional selection. The closed filter shapes of females were also consistent with stabilizing rather than directional selection (Figs. 1, 2). Indeed, the observation that the distribution of the chirp period was well beyond the range encompassed by female preference was more enigmatic (Fig. 2c). Without further knowledge about the behaviour and habitat of the *Steppengrille*, explanations remain presently elusive. It is conceivable, however, that females may avoid single males and search for aggregations in which shorter chirp periods arise by interference between singing males. Alternative explanations arise from the fact that the *Steppengrille* was kept in culture by breeders for more than 30 years. Breeders may have placed selection pressure on low singing activity, to satisfy customers. By that softly singing males with short chirps and long chirp periods may have been favoured. Female selectivity would have remained unaffected by this selection process unless there was genetic coupling between the mechanisms for song generation and song recognition ([30]). If there was selection imposed by breeders, the prominent divergence between male song and female preference was the result of domestication and may serve as a further example of rapid evolution of song traits ([31]). Alternatively, the mismatch of the signal and its recognition on the long time scale could also originate from a founder effect, when the *Steppengrille* was taken into culture.
